# 正性调节区锌指蛋白14的研究进展

**DOI:** 10.3779/j.issn.1009-3419.2016.02.06

**Published:** 2016-02-20

**Authors:** 玉栋 韩, 强 林

**Affiliations:** 200080 上海，上海交通大学附属第一人民医院胸外科 Department of Thoracic Surgery, Shanghai General Hospital, Shanghai Jiao Tong University, School of Medicine, Shanghai 200080, China

**Keywords:** PRDM14, 表观遗传学, 肿瘤, PRDM14, Epigenetic, Neoplasm

## Abstract

正性调节区锌指蛋白14（PR domain zinc finger protein 14, PRDM14）是PRDM家族中的重要成员，PRDM14基因对维持细胞的完整性和控制细胞的分化、生长及凋亡起着关键作用，在原始生殖细胞的形成、干细胞全能性的维持和其他组织器官的形成中都发挥了重要作用。PRDM14具有1个PR结构域和6个锌指结构，PRDM14参与了组蛋白的去乙酰化及甲基化过程，通过启动子区甲基化水平的改变参与肿瘤的形成。PRDM14异常甲基化能够引起染色质结构、DNA构象及DNA与蛋白质作用方式的改变，使基因的转录和表达受抑制，这些改变引起了肿瘤的发生、发展及转移。本文根据国内外发表的相关文献对PRDM14的研究现状进行综述。

## Abstract (en)

PR domain zinc finger protein 14 (PRDM14) is an important member of the PRDM family, PRDM14 plays a key role in the maintenance of cell integrity and differentiation, growth and apoptosis of the cell. It also plays an critical role in the formation of primordial germ cells, the maintenance of the totipotency of stem cells and the formation of tissues and organs. PRDM14 bears a single PR domain and six tandemly repeated zinc fingers, which is involved in the process of the deacetylation and methylation of the histone, and is involved in the formation of tumor trough the change level of methylation in the promoter region. The abnormal methylation of PRDM14 can change the chromatin structure, DNA conformation and the interaction mode of DNA and protein, it can suppress transcription and expression of the gene, which caused the occurrence, development and metastasis of tumor. The research progress of PRDM14 is reviewed based on the relevant literatures published in China and abroad.

PRDM源于N端保守的结构域，即PR结构域。PR结构域最初是在视网膜母细胞瘤蛋白质结合的锌指蛋白1（retinoblastoma protein-interacting zinc finger protein 1, RIZ1）和正性调节区Ⅰ-结合因子1（positive regulatory domain Ⅰ-binding factor 1, PRDI-BF1）两个蛋白中发现，因而被命名为PR（PRDI-BF1-RIZ1 homologous）domain^[1]^。*PRDM*基因家族大多数是抑癌基因，PRDM基因家族与人类许多疾病的发生发展密切相关^[2]^。*PRDM*基因源于多细胞动物，首先在脊椎动物中进行扩展，然后在灵长类动物中进一步复制^[3]^。到目前为止，已经在人体组织细胞中发现17个*PRDM*基因家族成员，分别命名为PRDM1-17^[3]^，PRDM家族的已知基因基本定位在肿瘤细胞中缺失的染色体区域。PRDM蛋白是Ca^2+^依赖磷脂结合蛋白，在许多生理和病理过程中都发挥着重要作用。这些蛋白的C-末端包含四个由氨基酸残基组成的重复序列，N-末端是蛋白修饰位点和作用位点。

## 1 PRDM简述

### 1.1 PRDM蛋白的结构特点

PR结构域和不等数量的锌指结构是PRDM家族的两个显著特点，锌关节也出现在部分PRDM蛋白中。

PR结构域是一段同源域，长约130个氨基酸，基本上位于蛋白质的N端。该结构域中有20%-30%的氨基酸与SET（Su(var)3-9, E(z) and trithorax）结构域一致^[4]^。在大多数SET结构域蛋白中已经检测到组蛋白甲基转移酶（histone methyltransferase, HMT）活性^[5]^，但是PR结构域具有组蛋白甲基转移酶活性的只有PRDM2、PRDM8和PRDM9^[6]^，有些PRDM蛋白（不具有HMT活性）可与一些酶作用，这些酶能够催化组蛋白甲基化，有些PRDM蛋白催化染色体翻译后修饰，在基因表达的表观遗传调控中发挥了重要作用。

基本上所有的PRDM家族成员都具有数量不等的锌指结构（除PRDM11外）^[7]^。PRDM与脱氧核糖核酸（deoxyribonucleic acid, DNA）和蛋白相互作用主要由锌指结构介导，已证实PRDM1、PRDM3、PRDM4、PRDM5、PRDM9、PRDM14和PRDM16能够利用锌指结构与DNA进行序列特异性的结合，组蛋白修饰酶的募集也可以通过锌指结构实现^[8, 9]^。

锌关节是一段长约20个氨基酸基序，锌关节能结合锌离子主要是因为含有半胱氨酸和组氨酸。PRDM4、PRDM6、PRDM7、PRDM9、PRDM10、PRDM11和PRDM15中都存在锌关节，锌关节可能参与蛋白-蛋白相互作用，位于PR结构域之前^[10]^。

### 1.2 PRDM蛋白的作用特点

国内外研究证实，PRDM蛋白家族行使功能的方式主要有以下三种。有些PRDM蛋白有内在的组蛋白甲基转移酶（histone methyltransferase, HMT）活性，这些PRDM蛋白可以对靶基因的染色质结构进行直接修饰，以此来实现基因的表达调控^[11]^。PRDM2和PRDM8主要是通过催化组蛋白H3K9的二甲基化（H3K9me2）来抑制基因表达，而基因表达的激活主要通过PRDM9催化H3K4的三甲基化（H3K4me3）来实现^[12]^。哺乳动物异染色质完整性的维持则依靠PRDM3和PRDM16催化的H3K9me1来实现^[13]^。

染色质编辑器是能够修饰染色质结构进而控制基因表达的蛋白^[14]^，其主要作用是通过与部分PRDM蛋白的结合来修饰染色质，实现基因表达调控^[2, 9]^。PRDM家族募集的染色质修饰酶能够抑制基因转录，该功能主要通过修饰酶对染色质的修饰来实现，这些修饰酶主要包括组蛋白去乙酰化酶、组蛋白乙酰转移酶及组蛋白甲基转移酶等。

除了上面两种作用方式以外，许多其他蛋白也能与PRDM蛋白相互作用^[8]^，如CtBP、Gata1、Jnk、Runx1、Smad1/Smad2、Smad3、Pu1、Pax2、Yy1、Fos、C/EBP β等，主要参与了信号转导及基因表达的调控。

## 2 PRDM14在干细胞中的作用

具有1个PR结构域和6个锌指结构的PRDM14蛋白是PRDM家族中重要的成员，PRDM14定位于染色体8q13.3上。通过对基因芯片的鉴定显示在未分化的人类胚胎干细胞中有PRDM14的表达^[15]^，PRDM14通过调节表观遗传学重编程和潜在全能性的获得来影响生殖细胞的特化及发育^[16]^。

多项研究^[16-19]^发现PRDM14是在生殖相关细胞系中最早特异性表达的基因，主要在原始生殖细胞和某些多能性干细胞中表达，在进化过程中高度保守，该基因的主要作用是维持生殖细胞的发育及多能性。PRDM14在成体组织中不表达，仅在胚胎干细胞（embryonic stem cell, ESC）和胚胎生殖细胞（embryonic germ cell, EGC）中低表达^[19]^。

在生殖细胞发育过程中PRDM14有特异性表达。在小鼠囊胚原始细胞团（胚胎期第3.5天）中PRDM14有暂时而微弱的表达，大约在第5.5天PRDM14表达消失。之后在第6.5天PRDM14又在PRDM14阳性前体细胞中表达，一直在原始生殖细胞中持续表达至13.5天-14.5天，表达模式在雄性和雌性中没有差异^[20, 21]^。将小鼠的PRDM14敲除后小鼠胚胎生长没有异常，成体器官也正常，但是雌性和雄性均丧失生育能力，导致在卵巢和睾丸内没有发现生殖细胞^[22]^。PRDM14敲除小鼠的Sox2表达也受到影响，而Sox2是维持细胞多能性的重要转录因子；发育多能性相关蛋白3（也称为Stella）是原始生殖细胞特异性表达的基因，PRDM14敲除后该基因的表达也不能上调。PRDM14的缺失导致全能性恢复的关键蛋白不能表达^[23]^。

PRDM14能够影响早期生殖细胞的重编程。随着原始生殖细胞的发育，DNA甲基转移酶（DNA methyltransferase, DNMT）的活性下降，引起了DNA的去甲基化^[24]^。相反，在PRDM14缺失的原始生殖细胞中，参与合成DNMT的基因的表达开始上调，如Dnmt3b。在PRDM14缺失的原始生殖细胞中Uhrf1（ubiquitin-like, containing PHD and RING finger domains 1）基因表达升高，该基因是维持Dnmt3b甲基转移酶活性的重要因子^[25]^。有研究^[26]^表明，为了确保生殖系基因的表达和PGC体细胞程序的抑制，PRDM14抑制了DNA甲基化和细胞外信号调节激酶（extracellular regulated kinase, ERK）的活性，下调了成纤维细胞生长因子受体2（fibroblast growth factor receptor 2, FGFR2）的表达。PRDM14缺失的原始生殖细胞不能抑制组蛋白赖氨酸甲基转移酶Glp的活性，导致H3K9me2高水平表达，而H3K9me2在正常生殖细胞的第8.5天开始下调。这种表观遗传重编程对于生殖细胞保持潜在的全能性是非常重要的^[27]^。

在原始生殖细胞形成胚胎生殖细胞过程中，PRDM14发挥了重要作用，细胞多能性会随PRDM14缺失而丧失。原始生殖细胞在一定的培养条件下可去分化成为多能性胚胎生殖细胞。PRDM14阳性的小鼠胚胎原始生殖细胞能形成胚胎生殖细胞样克隆，而PRDM14阴性的小鼠胚胎原始生殖细胞则不能形成胚胎生殖细胞样克隆^[28]^。

## 3 PRDM14与肿瘤的关系

### 3.1 PRDM14与肺癌

肺癌的发生是多步骤的过程，涉及癌基因的激活和抑癌基因的失活。除了基因水平上的突变、缺失等，表观遗传学的改变，特别是DNA启动子区和CpG岛区域的异常甲基化是导致抑癌基因失活和癌基因激活的重要原因。由于表观遗传学的改变常发生于遗传学改变之前，因此在肺癌发生的早期就可能检测到抑癌基因和癌基因的异常甲基化，这对于肺癌的早期诊断具有重要意义。

*PRDM14*通过启动子区甲基化水平的改变参与肺癌形成^[2]^，甲基化通常发生在基因的启动子区和CpG岛，能够引起染色质结构、DNA构象及DNA与蛋白质作用方式的改变，使基因的转录和表达受抑制^[29]^。DNA的异常甲基化是癌基因和抑癌基因表达异常的重要机制，也是真核细胞基因组常见的表观遗传修饰。肿瘤发生相关基因启动子CpG岛异常甲基化是肿瘤发生早期的重要分子事件^[30]^。

PRDM14参与组氨酸的去乙酰化和甲基化过程，这可能是PRDM14参与肺癌发生发展的机制之一。研究证实PRDM14的表达水平随着肺癌分化程度的升高而升高，推测该基因可能在肺癌发生的早期起作用，促进细胞增殖。随着肺癌进展和分化不良，PRDM14蛋白表达下调，可能是由于PR结构域的CpG岛发生了甲基化进而抑制了PRDM14 mRNA的表达。研究显示PRDM14是致癌基因，转染*PRDM14*基因到A549细胞中明显促进细胞克隆形成及体内致瘤性，细胞增殖和迁移能力都得到了增强。甲基化焦磷酸测序显示PRDM14在早期肺癌组织中甲基化水平降低，mRNA表达量升高，而在癌旁组织甲基化水平升高，mRNA表达量降低，这与PRDM14在早期肺癌标本中的免疫组化结果吻合：癌组织高表达，癌旁组织低表达。说明PRDM14异常甲基化在早期肺癌的发生中起了关键作用，具体机制尚需进一步的研究。

国内学者^[31]^分析了非小细胞肺癌患者癌组织中PRDM14的表达水平，结果显示PRDM14在原发肿瘤样本和伴有淋巴转移的样本中表达量增加，肿瘤分化程度越高，PRDM14的表达量越高。最新一项研究显示，PRDM14通过细胞外基质降解促进肺癌细胞转移。在这项研究中，PRDM14感染的A549细胞迁移能力明显增强。基质金属蛋白酶（matrix metalloproteinases, MMPs）的功能受其组织抑制因子（tissue inhibitor of metalloproteinases, TIMPs）的调控，研究发现受PRDM14-shRNA转染的A549细胞MMP1表达上调，而TIMP1和TIMP2表达下调。MMP1上调和TIMP1/2的下调促进了细胞外基质的降解，间接促进了肿瘤细胞的迁移^[32]^。

### 3.2 PRDM14与乳腺癌

Nishikawa等^[33]^研究发现乳腺癌组织中PRDM14往往过表达，而PRDM14在正常乳腺组织里几乎没有表达。这些异常表达的PRDM14促进了肿瘤细胞的侵袭生长，降低了乳腺癌细胞对化疗药物的敏感性。有研究发现PRDM14的第二内含子DNA甲基化水平升高。抑制PRDM14的表达能诱导乳腺癌细胞凋亡，并增加其对化疗药物的敏感性，提示PRDM14可能是乳腺癌治疗的有效靶点。

PRDM14除与肺癌和乳腺癌有关外，还和宫颈癌等有关，且作用机制和甲基化相关^[34]^。

## 4 总结

PRDM14作为PRDM家族的重要成员，在调节胚胎干细胞潜在全能性的获得及表观遗传学重编程、影响生殖细胞特化和发育中发挥了重要作用^[35]^。PRDM14在肿瘤中的功能研究尚处于起步阶段，该基因异常甲基化与肿瘤发生、发展及转移的分子机制相关研究还不够深入，PRDM14在肿瘤干细胞及肿瘤耐药方面的研究尚少，尤其要深入研究其中的分子机制。PRDM14的表达和甲基化状态与肿瘤的临床病理、分期与转移复发的相关性还需要大样本前瞻性研究来阐明。
